# Barriers of organized cervical cancer screening in Albania and Montenegro

**DOI:** 10.1186/s12889-025-22535-4

**Published:** 2025-04-24

**Authors:** Marcell Csanádi, Kozeta Filipi, Alban Ylli, Bajram Dedja, Anila Bejko, Ivana Nikcevic Kovacevic, Jovana Vukovic-Lekovic, Milica Stanisic, Adrijana Vujovic, George Dennis Obeng, Inge M. C. M. de Kok, Zoltán Vokó, Orsolya Varga

**Affiliations:** 1 Center for Health Technology Assessment, Semmelweis University, Budapest, Hungary; 2 Syreon Research Institute, Budapest, Hungary; 3 Syreon Research Africa, Accra, Ghana; 4 Institute of Public Health of Albania, Tirana, Albania; 5 Albanian University of Tirana, Tirana, Albania; 6 University of Medicine of Tirana, Tirana, Albania; 7 University Hospital Centre, “Mbreteresha Geraldine”, Tirana, Albania; 8 Institute of Public Health of Montenegro, Podgorica, Montenegro; 9 Department of Public Health, Erasmus MC University Medical Center, Rotterdam, The Netherlands; 10 Department of Public Health and Epidemiology, Faculty of Medicine, University of Debrecen, Debrecen, Hungary

**Keywords:** Cervical cancer, Organized cancer screening, Southeastern Europe, Barriers of screening

## Abstract

**Background:**

Organized cervical screening is vital for preventing cervical cancer. However, many existing screening programs fail to achieve their full potential, as demonstrated by core performance indicators. There are barriers that hinder the implementation and reduce effectiveness of the programs. This article explores barriers of cervical cancer screening in two Southeastern European countries, Albania and Montenegro, aiming to inform targeted strategies to improve healthcare equity and outcomes for women.

**Methods:**

The barrier assessment followed the EU-TOPIA framework, designed to identify barriers to effective breast-, cervical-, or colon cancer screening. This approach relies on an iterative process performed by country representatives responsible for screening and researchers with expertise in screening program planning and evaluation. It includes three steps: comprehensive description of screening activities; identification of key barriers via a previously published tool; and comprehensive assessment of the identified key barriers.

**Results:**

The barrier assessment revealed shared challenges in cervical cancer screening in Albania and Montenegro. Both countries face difficulties in their invitation systems, limited outreach activities, and low participation rates. Fully integrated data systems at national level are absent, hindering program monitoring and data sharing. Financial constraints and resource limitations negatively affect program sustainability and quality, reducing public awareness and accessibility. Additionally, neither country has comprehensive up-to-date long-term strategies to support prevention and early detection efforts.

**Conclusions:**

Our study underscores the importance of addressing organizational barriers in cervical cancer screening to improve program effectiveness and accessibility. Aligning screening practices with EU and WHO standards is crucial for Albania and Montenegro as prospective EU members. Lessons from international best practices, such as integrating IT systems, employing multi-channel outreach strategies, and adopting legally supported long-term policies, offer actionable pathways for improvement. Policymakers should prioritize sustainable funding, centralized systems, and innovative approaches to overcome structural challenges.

**Supplementary Information:**

The online version contains supplementary material available at 10.1186/s12889-025-22535-4.

## Background

Organized cervical screening programs are of paramount importance in the early detection and prevention of cervical cancer. These programs significantly contribute to the reduction of morbidity and mortality associated with the disease [1]. Regular screening allows for the identification of precancerous lesions, thereby enabling timely intervention that can prevent the progression to invasive cancer [2]. Available evidence indicates that effective screening can reduce the incidence of cervical cancer by up to 80% when conducted at recommended intervals and with high quality [3]. The implementation of organized screening program ensures that a larger population is reached, particularly vulnerable groups, who may otherwise have limited access to healthcare services [4].

In the European Union, guidelines recommend that women aged 30 to 65 undergo screening every three to five years, depending on the type of test used, to maximize early detection and treatment opportunities [5]. In addition, it clearly states that opportunistic screening, which takes place in clinical settings and depends on the initiative of the individual woman or her doctor, should be discouraged, while a well-organized screening programme must reach high population acceptance and coverage with ensuring high quality at all levels [6].

Although, the importance of cervical cancer screening is widely accepted, considerable differences exist across countries’ screening guidelines, even among comparable systems, while methods of monitoring are also heterogenous and systematic data collection with regular assessment is often not established [7]. In addition, many programs fail to achieve their full potential, as demonstrated by core performance indicators. Globally, the participation rates in cervical cancer screening programs remain low. Estimates indicate that approximately 32% of women between the ages of 30 and 49 have undergone screening within the past five years, as of 2019 [8]. In the European Union, there is a considerable variation in participation rates among member countries. Some countries report rates as high as 80%, while others have rates less than 40% [8]. The Cancer Screening in the European Union report found that there were considerable variations even in the invitation rates among countries [9].

In order to maximize the benefits from cervical cancer screening, a holistic analysis of the screening barriers is required [10]. In the context of the Southeastern European countries, it is of particular importance to address these barriers due to the influence of socio-economic factors and the limitations of healthcare infrastructure, which can significantly impact women’s access to preventive services. By focusing on these issues, stakeholders can implement targeted strategies that improve healthcare access and equity, ultimately leading to better health outcomes for women in Southeastern Europe.

Two representative countries for the cervical cancer screening situation in Southeastern Europe are Montenegro and Albania. The organized cervical cancer screening program in Montenegro was initiated on July 18th, 2016, as a pilot project in the municipality of Podgorica, targeting women aged 30–34. Since February 1st 2018, the screening program has been conducted at the national level among the insured women of the Health Insurance Fund of Montenegro, who are registered with a gynecologist. According to the National Organized Cervical Cancer Screening Program, currently the target population is women aged 30–50, with the aim to extend this to 30–64 years [11]. The primary screening test is the molecular Human Papillomavirus (HPV) test, based on the detection of high-risk HPV genotypes DNA from cervical swab samples. The duration of one screening round is five years. The primary screening tests are conducted by chosen gynecologists for women in all health centers in Montenegro. After a positive HPV DNA test, the triage test is liquid-based cytology. Recently, it has become possible that during a single visit to the gynecologist, a cervical swab is collected for primary testing, and if the primary test result is positive, the cytology can be conducted from the same sample without requiring a follow-up visit. There has been no published evaluation of the program since its establishment.

In 2010, Albania began developing more structured policies, culminating in the first National Cancer Control Plan (2011–2020). The National Cervical Cancer Screening Program in Albania is governed by a ministerial decree published in 2019. The program uses high-risk HPV testing as primary screening examination. The initial program targeted women 40–50 years old, with a plan to be extended to 35–60 years of age [12]. Under the program, all primary screening tests and further examinations are to be provided free of charge at the point of care, regardless of the patients’ health insurance status. The Institute of Public Health coordinates the program and provides the technical expertise and logistical support when needed. At regional level the coordinators are responsible for local coordination and management; receiving test kits from Institute of Public Health and distributing them to every health center, then collecting the samples/information and sending to the Institute of Public Health, and delivering laboratory results to health centers. The latest evaluation report about the program was conducted in 2020 after the first year of implementation with WHO support [13].

The objective of this article is to present the barriers of organized cervical cancer screening in Southeastern European countries by investigating two countries: Albania and Montenegro.

## Methods

The barrier assessment was conducted according to the approach that has been developed in the EU-TOPIA project, an EU funded project. The Barriers to Effective Screening Tool was developed to identify the barriers to effective breast, cervical and colorectal cancer screening [14]. It was proven to be an applicable tool as it was used in various countries and cancer types [10, 14, 15]. It is based on an approach that categorizes potential barriers according to the subsystems of screening: identification of population at risk; generation of knowledge and effectiveness; maximization of uptake (informed participation); operation of the program; maximization of follow-up; Treatment [16]. The barriers in the tool were defined according to a conceptual framework that includes health system barriers (i.e. availability of resources, affordability and acceptability of health services), capability barriers (i.e. knowledge or skills to implement effective screening programmes) and intention barriers (i.e. motivations of providers to achieve effective screening) [17].

Barrier assessment was carried out through a step-by-step process using standardized templates that are publicly accessible [18]. Details on this process is available on the website of the EU-TOPIA-EAST project in a form of a publicly available webinar [19]. We drew upon multiple sources of information from Albania and Montenegro, including published literature, presentations, policy documents, guidelines and expert opinions. Most relevant materials regarding screening activities were local documents, as there were no published studies in English on cervical cancer screening from these countries. Throughout each stage of the barrier assessment, an iterative process was facilitated between country representatives and researchers with expertise in screening program planning and evaluation. Country representatives included program coordinators and researchers from the Institute of Public Health of Montenegro and the Institute of Public Health of Albania. Regular online meetings and continuous correspondence supported this collaborative process in the framework of the EUTOPIA-EAST project. The barrier assessment spanned from August 2024 to October 2024, with each phase of the process detailed below.

Step 1: Comprehensive Description of Screening Activities.

The initial step involved a thorough description to contextualize and outline the context cervical cancer screening activities in Albania and Montenegro. We utilized a structured document for characterizing cancer screening programs based on the following domains: (1) historical overview; (2) patient/individual pathway; (3) data collection and IT infrastructure; (4) organizational background including stakeholders and legal framework; (5) capacities and available resources; and (6) individual perception and cultural background.

Step 2: Identification of Key Barriers.

The second step involved identifying key barriers using the Barriers to Effective Screening Tool. This step aimed to enable the representatives to conduct a self-assessment of their cervical cancer screening program to identify the most significant barriers. This involved scoring a list of potential barriers from two perspectives: their impact on the effectiveness of the screening program and their effect on equity. The outcome was the formulation of a priority list, which highlighted the three most critical barriers that required attention in Albania and Montenegro.

Step 3: Comprehensive Assessment of the Identified Key Barriers.

In the third step, we conducted a detailed assessment of the prioritized barriers to provide an in-depth understanding of each barrier, and the challenges associated with overcoming them. We employed a structured document with questions related to four main topics: (1) historical context of the barrier; (2) capabilities and resources influencing the barrier; (3) stakeholders’ perspectives; and (4) available knowledge, data, and monitoring information related to the barrier. The template incorporates a series of targeted questions designed to elicit detailed responses concerning each identified barrier.

## Results

As the screening systems were briefly described in the Introduction section, here we present the in-depth description of the identified key barriers to the cervical cancer screening program ranked according to their priorities. The following key barriers were identified, see Table 1.

**Table 1:** Identified key barriers in Albania and Montenegro

	Montenegro	Albania
Barrier ranked #1	Inadequate capacities for improving the invitation system and participation rate	Inadequate procedures for the invitation of woman eligible for screening
Barrier ranked #2	Absence of an integrated screening registry for cervical cancer	Insufficient financial resources to operate the screening program
Barrier ranked #3	Lack of up-to-date, long-term strategy for prevention and early detection of cervical cancer	Lack of effective information systems and technology in cervical cancer prevention

### Summary of key barriers identified in Montenegro

#### Inadequate capacities for improving the invitation system and participation rate

The Institute of Public Health of Montenegro (IPHMNE) coordinates a screening program for women by using data from the Health Insurance Fund and the Central Population Register to identify eligible participants. While finding the target population is straightforward, challenges arise from outdated contact information and inefficient outreach methods. Nurses, part of gynecologists’ teams, are responsible for inviting women to participate in screening program. However, due to high workloads, limited resources, and low financial incentives, their ability to reach women effectively is constrained. Contact information is often outdated since it is only refreshed during healthcare visits, and many women aged 30 to 50 frequently change phone numbers or opt for private gynecological care. Data privacy laws prevent the acquisition of updated contact details from mobile operators, and mail outreach has shown limited effectiveness. Although an initiative was proposed to establish a call center focused on contacting participants to relieve the burden of nurses, however, this has not yet been implemented.

#### Absence of an integrated screening registry for cervical cancer

The daily operation of the screening program faces major challenges, primarily due to the lack of a dedicated screening registry. This registry is critical for monitoring and evaluating the program effectively. Additionally, the program is hindered by the use of two separate data entry systems: one for screening data, and one for the gynecological, oncological, and pathology records at the Clinical Center of Montenegro. Without integration between these systems, healthcare providers, who are already overburdened, must enter data into both, which leads to inefficiencies and reduces compliance. The lack of connectivity between the two platforms significantly impedes data flow to the IPHMNE, preventing it from accessing essential information for program monitoring. Consequently, while critical data exists across both systems, the effective usage is limited, compromising the quality of program oversight.

#### Lack of up-to-date, long-term strategy for prevention and early detection of cervical cancer

Currently there is a lack of a up-to-date and long-term comprehensive strategy and for screening programs. The current strategy could benefit from updates and enhancements. While there are guides and updated algorithms for conducting screenings, the absence of a long-term strategy for prevention and early detection of cervical cancer hampers the program’s quality and effectiveness. Frequent changes in health ministry leadership make it challenging to develop new and consistent strategies and policies.

### Summary of key barriers identified in Albania

#### Inadequate procedures for the invitation of woman eligible for screening

The introduction of HPV testing in 2019 marked a turning point in Albania. However, participation of eligible woman is constrained by limited outreach, low funding, and logistical issues. With screening largely restricted to insured women registered with a family doctor, many eligible individuals, especially from rural or marginalized groups, remain unreached by the screening program. While hypothetically all invited women participate, the protocol is inconsistently applied. In many cases informal routes and connections are prioritized to invite woman for screening, including the relatives, companions or staff members, leaving significant gaps in reaching the total intended age group. This inadequate procedure to invite individuals meaningfully contributes to the observed 30% screening coverage of the target population of women aged 40–49. Several stakeholders are affected and actually could be capable to overcome the barrier, however, currently there is a lack of knowledge among these stakeholders on implementing coordinated action to move towards solving the issue.

#### Insufficient financial resources to operate the screening program

Key challenges affecting cervical cancer screening in Albania include persistent funding gaps, as limited government funding and reliance on external donors make it difficult to establish and maintain the program. Additionally, high costs of screening and follow-up care pose significant barriers, especially in rural areas, where access is already limited. The lack of adequate funds also hampers public awareness efforts, reducing the reach and impact of education on cervical cancer prevention. Furthermore, underfunded training and infrastructure constrain the quality and accessibility of services, limiting the overall effectiveness of the program. While building partnerships and securing long-term investment would support sustainability, these efforts have so far been insufficient. Notably, without additional resources and careful planning, Albanian health authorities face significant challenges in expanding and improving cervical cancer screening services. Advocacy and policy development have been pursued, primarily by local and international organizations, aiming to influence policymakers for better resource allocation for cancer prevention. However, there are no year-round activities in place to sustain support for the cervical cancer screening program. The program’s budget has remained unchanged for years and lacks flexibility, limiting its ability to expand services or cover additional costs. The limited funding for Albania’s cervical cancer screening program creates challenges for a wide range of stakeholders. Government agencies struggle to develop effective screening policies, while the Health Insurance Fund cannot fully support preventive services. Key organizations, including the National Cancer Control Program and the Institute of Public Health, are constrained in their outreach, research, and policy work.

#### Lack of effective information systems and technology in cervical cancer prevention

The current monitoring system for cervical cancer screening in Albania is incomplete, only covering primary testing and leaving follow-up care largely undocumented. There is no centralized IT system to monitor treatment outcomes or track patient data, making it difficult to assess the overall effectiveness of the screening program. Additionally, individual reporting forms are not automatized, leading to inefficiencies and potential data loss. There has been no investment to improve the technology on data collection. The process is ineffective due to the required time and effort at data entry and data analysis. Addressing barriers to information systems and technological advances in cervical cancer prevention requires a multifaceted approach that combines innovation, training, community engagement, and policy development. Basically, all stakeholders are impacted by this barrier. International organizations such as WHO and the EU have previously provided support through policy advice and financial aid. However, better collaboration between these groups and the introduction of more robust systems for coordination are necessary to make meaningful progress. Finally, the lack of adherence to protocols of screening also contributes to this barrier. Many professionals working within the system have not received updated training on new technologies or best practices for HPV screening and follow-up.

## Discussion

Our study provides an in-depth look into the barriers of cervical cancer screening for two countries that serve as an example in many countries in Southeastern Europe. We applied widely published and publicly available tools and approaches. The understanding of these barriers is a key element to maximize the potential of early detection of cancer. Once these are clearly described, policy actions can be better tailored and targeted. This step-by-step process has been developed by the EU-TOPIA and the subsequent EU-TOPIA-EAST project [19], and is part of the country specific roadmap development to improve cancer screening programs in Europe. The approach has, amongst others, been successfully used in Montenegro to improve the colorectal cancer screening system [20].

### Similarities and differences across countries

The barrier assessment presented in this study revealed similarities and differences between the two investigated countries. Both Montenegro and Albania encounter obstacles in the implementation of cervical cancer screening programs, including the limited effectiveness of invitation systems and the scarcity of outreach activities, which collectively result in lower participation rates and less effective cancer prevention. Furthermore, both countries are lacking fully integrated data systems at national level, which impedes the efficient monitoring of programs and the sharing of data through the information systems. Financial constraints and limited resources have an adverse impact on the sustainability and quality of their programs, which in turn limits public awareness and accessibility. Furthermore, both countries lack comprehensive, long-term strategies to guide their prevention and early detection efforts.

However, to address these challenges, the two countries have opted for disparate strategies. The difficulties encountered by Montenegro in the invitation process are attributable to the outdated nature of the contact information available and the high workloads experienced by nurses. On the other hand, in Albania, the reliance on inconsistent and informal outreach strategies has the effect of excluding a significant proportion of eligible women.

Montenegro has two distinct, unintegrated data systems, whereas Albania lacks a centralized system entirely, thereby limiting its capacity to track follow-up care. In terms of financial resources, Montenegro is constrained internally, whereas Albania is heavily reliant on external donors, which has an adverse impact on the sustainability of its programs.

The challenges encountered in the implementation of cervical cancer screening programs in the cases of this study are reflective of the broader barriers that are observed at the global level as well. Governments frequently prioritize treatment over prevention due to the limited resources available and the visibility of advanced diseases. This results in underfunded screening programs and fragmented healthcare systems. Deficits in healthcare infrastructure, including outdated IT systems and inadequate registries impede efficiency. Overburdened healthcare workers and political instability, such as frequent leadership changes, disrupt long-term strategies [21]. Previous studies have shown that cultural stigmas and low awareness further reduce participation, particularly in rural and marginalised communities where access is limited [22].

### Stakeholder involvement

In the future, stakeholder involvement to resolve current challenges are crucial. In Montenegro key stakeholders—such as the Ministry of Health and the IPHMNE—could collaborate to update contact information and revamp outreach methods; for example, establishing a dedicated call center could streamline invitations and boost screening participation among eligible women. Montenegro’s cervical cancer screening program currently struggles with operational inefficiencies due to the lack of a centralized registry and the absence of integration between two separate data systems (for screening data and for related clinical records), which forces overburdened healthcare providers to duplicate data entry and impedes effective monitoring. To overcome these obstacles, Montenegro should first establish a comprehensive screening registry through robust stakeholder collaboration and then implement an integrated solution for seamless data sharing, thereby enhancing data management and overall screening efficiency. As an EU accession candidate, Montenegro must align its screening programs with EU standards by developing a formal new, up-to-date strategy that sets clear goals and a long-term action plan with measurable outcomes, while also addressing policy discontinuity caused by frequent changes in health ministry leadership. In Albania, several stakeholders—including the Ministry of Health, local governments, the National Cancer Control Program, and the Institute of Public Health—are well positioned to overcome current barriers by prioritizing cervical cancer screening in policy and legislative frameworks, improving targeted outreach, and promoting educational initiatives. Additionally, the Albanian Medical Association can contribute by enhancing community outreach, training, and service quality. However, a current lack of coordinated action among these stakeholders limits progress, underscoring the need for improved collaboration and knowledge sharing to effectively overcome these barriers.

### Learning opportunity from other countries

The approach of learning from best practices in cervical cancer screening has demonstrated success internationally, offering solutions to barriers in countries such as Albania and Montenegro. Multi-channel outreach strategies, as implemented for instance in Sweden, combine personal invitations, reminders, and digital registries to improve participation rates [23]. Sweden’s organized screening program integrates opportunistic tests into its centralized system, enhancing coverage and reducing inefficiencies​. Australia’s program highlights the importance of legally supported, long-term strategies [24]. Technological integration, seen in Estonia and Brazil, improves program efficiency. Centralized IT systems and automated workflows likely streamline data management and reduce the administrative burden on healthcare providers [25, 26]. Training healthcare professionals in new technologies ensures adherence to updated screening protocols​. Both countries can benefit from collaboration, innovation, and strategic policy development to expand cervical cancer prevention efforts and align with international standards.

A systematic review on cancer screening for middle and low-income countries revealed that, while a lack of awareness, embarrassment, lack of family support and cost of screening were identified as important barriers, the fear of a cancer diagnosis due to a lack of financial resources for subsequent tests and treatment was the most significant barrier, exerting a pervasive influence on the other barriers [27]. In Albania and Montenegro, where universal healthcare access is available, the identified barriers are primarily organizational in nature and require a different comprehensive approach to their resolution. In their capacity as prospective members of the European Union, Montenegro and Albania are obliged to align their screening programs with the standards set out by the EU and the WHO [28].

## Limitations

Our study has some notable limitations. Firstly, we relied on publicly available information and in many occasions, on the experience of program coordinators and researchers working at the national public health institutes. We had no opportunity to widen the scope of stakeholders within this research. Therefore, in the future it would be important to validate the information presented in the article by other key stakeholders. Secondly, we collected mostly qualitative information and provided a narrative synthesis that was developed in collaboration with the country representatives. Due to the limited availability of public data on screening, we could not complement our qualitative results with quantitative evidence. Finally, to make this study feasible, we had to limit our barrier assessment to the three most important ones. However, it should be acknowledged that in both countries there should be also other barriers not captured in this study.

## Conclusion

Eventually, it is our hope that our paper provides a solid basis for tailoring policy actions in the investigated countries by which these barriers can be overcome in the future. These can serve as an example for countries facing similar challenges. In addition, our approach has proven to be applicable in various jurisdictions already, so we advocate to perform similar assessment in countries where the organized cancer screening programs are facing difficulties to deliver the expected benefits to the society. Only by solving these barriers we can reduce the cervical cancer burden, and make a first step towards global cervical cancer elimination [29].

## Electronic supplementary material

Below is the link to the electronic supplementary material.

Supplementary Material 1

## Data Availability

The datasets used and/or analysed during the current study are available from the corresponding author on reasonable request.
