# Supplementary material for: Barriers of organized cervical cancer screening in Albania and Montenegro
Source: BMC Public Health. 2025 Apr 24;25:1533. doi: 10.1186/s12889-025-22535-4 (PMC12020179; doi:10.1186/s12889-025-22535-4)
Supplement: Supplementary file 1 — Supplementary Material 1 [file 12889_2025_22535_MOESM1_ESM.docx]

**COMPREHENSIVE ASSESSMENT TO UNDERSTAND**

**THE CONTEXT AND THE ENVIRONMENT OF IDENTIFIED KEY BARRIERS**

Introduction and aims

In work package 3 of the EU-TOPIA-EAST project, road maps to improve cancer screening programmes are developed, which include a detailed description of screening activities, a barrier assessment, and an outline for action plan for implementation. This document aims to provide a structured framework to perform the activity about the comprehensive assessment of the most important barriers of the screening programme in the investigated country.

***This activity should be considered as an important step in the road map development process***. The completed document provides a detailed explanation of each barrier based on structured questions, which eventually feeds into the action plan development in the EU-TOPIA-EAST project. This comprehensive overview of the barriers contributes to outlining appropriate and feasible actions and reveal the detailed problems that make it difficult to actually overcome the barriers.

The document is structured according to the identified barriers, therefore it is prerequisite to first complete the EU-TOPIA Barrier Assessment Tool, to identify the most important barriers in the investigated country. The tool, can be downloaded in an Excel file format from the following link: <https://eu-topia-east.org/workpackages/wp3-road-maps-to-improve-cancer-screening/>

The comprehensive assessment of each identified barrier consists of 4 main chapters:

- Historical context of the identified barrier
- Capabilities and resources of the programme that influence the barrier
- Stakeholders’ perspectives on the barrier
- Available knowledge, data, and monitoring of the barrier

The questions in these chapters should be completed by using various available data sources and considering the ‘Comprehensive description of the screening activities’ document, that also should developed as part of the road map development process in the EU-TOPIA-EAST project. Data sources may include published scientific papers, other publicly available documents (i.e., grey literature), presentations or expert opinion.

The document starts with a background information section, where some details should be provided based on a list of questions. Then it continues with the comprehensive assessment of the most important barrier, which was identified in the Barrier Assessment Tool, based on the 4 main chapters. This is followed by the other two identified key barriers. Questions within the chapters are asked in a tabulated format. The tables should be completed by marking the answer (i.e., yes / no), then a free text explanation should be given according to the question-specific instructions.

***Please complete the areas coloured with light grey! If a question is not applicable or not relevant, please mark with N/A***.

It is recommended to complete the document in an iterative process between the country-representative and the Syreon Research Institute team, which is the consortium partner in the EU-TOPIA-EAST that is responsible for this task. Therefore, before the start of this activity and during or after completion, it is recommended to contact the team via e-mail on the following address: [eu-topia@syreon.eu](mailto:eu-topia@syreon.eu)

Background information

Please name the country for which you complete this document!

| Montenegro |
| --- |

Please name the cancer site for which you complete this document!

| Cervical cancer |
| --- |

Please list the most important barriers that were identified in the Barrier Assessment Tool!

| Barrier #1: | Inadequate capacities for improving the invitation system and participation rate |
| --- | --- |
| Barrier #2: | Absence of an integrated screening registry for cervical cancer |
| Barrier #3: | Lack of strategy for prevention and early detection of cervical cancer |

Please describe your position or your relationship with the screening programme (programme coordinator / researcher / policymaker / clinician / patient)!

| Programme coordinator |
| --- |

Please mark the date when you completed this barrier assessment document (year and months)!

| November 2024 |
| --- |

| **Barrier #1:** | Operation - Inadequate response for improving the invitation system and participation rate |
| --- | --- |

**CHAPTER 1: HISTORICAL CONTEXT OF THE IDENTIFIED BARRIER**

**Question 1. Can you identify any reason(s) responsible for the emergence of the barrier regarding the history of the screening program in your country?** (Mark your answer with an x.)

| No |  | Move to Question 2! |
| --- | --- | --- |
| Yes | x | Describe the reason(s) below! |

| Please, describe any historical reason that directly or indirectly led to the emergence of the barrier in the past (e.g., attributes, events).  *If there are multiple reasons, please describe all of them and define the most important one that led to the emergence of the barrier.* | If you are aware of any information source related to the reason(s), please, list here. |
| --- | --- |
| Historically, a significant number of women lacked health insurance, which limited their participation in the screening program. At that time, there was a discrepancy between the number of women registered in the Central Population Register and the number of women eligible for the screening program. However, this issue was effectively resolved with legislative changes made in 2022.  The Institute for Public Health of Montenegro (IPH MNE) utilizes data from the Health Insurance Fund (HIF), which automatically retrieves information for each insured individual from the Central Population Register (CPR). This process establishes an indirect link between the IPH MNE and the CPR, enabling the identification of the target population crucial for screening initiatives.  While there is no issue with identifying the target population, challenges arise due to outdated contact information and the method of communication used to reach participants.  Currently, medical nurses from the doctors’ teams (gynecologists) are responsible for contacting participants to schedule screenings. They do not have dedicated phone lines for this purpose and often use either official numbers from the Health Center or their personal phones. Due to their high workload, these nurses struggle to find time to make calls, as there is no specific time allocated for outreach; they fit this task into their daily schedules when possible. Low financial compensation combined with a heavy workload makes it challenging for them to feel motivated to reach out to participants, especially considering the effort required to encourage women to attend screenings.  Contact information, including phone numbers, is only updated when a women visits a healthcare provider. This means that the information about the target population is refreshed annually; however, to obtain accurate phone numbers, women must provide their updated information during their interactions with the healthcare system (choosen gynecologist). A significant number of women do not visit their gynecologists regularly, resulting in outdated phone numbers on their records. Since the target population consists of women aged 30 to 50, many frequently change their phone numbers and do not update their information in their medical files. Additionally, some women may never visit their gynecologists at the Health Center; they may either avoid gynecological care entirely or choose to go to private facilities that are not linked to the Health Centers. Consequently, although these women are included in the call list, there is no access to their contact information.  These factors collectively hinder effective communication and participation in the screening program. |  |

**Question 2. Are you aware of any activities or attempts that aimed to overcome the barrier in the past?** (Mark your answer with an x.)

| No |  | Move to Question 3! |
| --- | --- | --- |
| Yes | X | Describe the activities or attempts below! |

| Please, describe the activities and attempts that aimed to overcome the barrier in the past (i.e., how, when, by whom, where).  *Explain why these attempts failed / did not fully solve the problem.* | If you are aware of any information source related to the activities, please, list here. |
| --- | --- |
| Several attempts were made to get phone numbers from the mobile operator for the screening participants. Due to the law on the protection of personal data, this is not possible.  What can be used are publicly available directories of mobile operators, but very often they do not contain updated phone numbers.  Additionally, in the context of a past project, one of the activities involved contacting participants by sending letters to their home addresses. However, this method proved to be less effective for several reasons: many streets in Montenegro do not have designated house numbers, individuals sometimes do not reside at the addresses where they are officially registered, and there are numerous cases of people with the same name and surname living on the same street without clear identification of their home address.  An initiative was proposed to establish a call center focused on contacting participants, but this has not yet been implemented. While the creation of a call center would not alone resolve the issue of missing phone numbers for women, it would significantly alleviate the workload for the medical nurses who are currently responsible for making these calls.  Additionally, there is an e-Health application through which citizens could update their contact information, making it visible within the healthcare system. However, this application has not been widely adopted. |  |

**Question 3. Can you identify any relevant changes in the health care system or in other systems (political, economic, legislative) that effected the barrier in the past?** (Mark your answer with an x.)

| No |  | Move to Question 4! |
| --- | --- | --- |
| Yes | x | Describe the changes below! |

| Please, describe these changes that directly or indirectly affected the barrier.  *Explain whether the changes supported to overcome the barrier or made the problems more difficult.* | If you are aware of any information source related to the changes, please, list here. |
| --- | --- |
| In the previous few years, the minister of health and his associates have been changed several times. So we have again had to inform the decision makers about the barriers in the screening program.  Because of these changes, the law on health protection regulating the screening program has still not been adopted. |  |

**CHAPTER 2: CAPABILITIES AND RESOURCES THAT INFLUENCE THE BARRIER**

**Question 4. Does the current legislative, political and economic environment allow or support to overcome the identified barrier?** (Mark your answer with an x.)

| Yes |  | Move to Question 5! |
| --- | --- | --- |
| No | X | Describe the reason(s) below! |

| Please, describe any reason from the legislative, political and economic environment point of view that does not allow or support to overcome the identified barrier.  *If there are multiple reasons, please, describe all of them and define the most important one.* | If you are aware of any information source related to the reason(s), please, list here. |
| --- | --- |
| This barrier could be addressed by changing legal regulations to allow access to mobile operators' databases for the purposes of public health activities related to the organized screening program. This would enable the matching of women with their phone numbers based on their unique identification numbers. Additionally, outreach by the teams of choosen doctors could be facilitated in several ways: by hiring new staff, identifying additional time slots for calls, covering mobile phone expenses, increasing compensation for those involved in the screening program calling process, or establishing call centers. Furthermore, it is essential that all these activities be implemented by the Ministry of Health, as the institution that would have the authority to mandate such initiatives. |  |

**Question 5. Are there any major obstacles from the human resource point of view to overcome the barrier?** (Mark your answer with an x.)

| No |  | Move to Question 6! |
| --- | --- | --- |
| Yes | x | Describe the obstacles below! |

| Please, describe the human resource obstacles. | If you are aware of any information source related to the obstacles, please, list here. |
| --- | --- |
| There is no trained staff specifically designated for contacting participants in the screening program. This responsibility falls to nurses who regularly work in gynecological ambulances as part of the choosen gynecologist's team. They are often overwhelmed with their regular duties and can only engage in screening activities when time permits. Their motivation is low, as minimal funding is allocated for these efforts, and they lack dedicated phones for this purpose. Moreover, it requires significant time to effectively encourage the women they call to attend for screening. |  |

**Question 6. Are there any major obstacles from the technological resources or capacities point of view to overcome the barrier? (IT system, screening technologies and devices, equipment etc.)** (Mark your answer with an x.)

| No |  | Move to Question 7! |
| --- | --- | --- |
| Yes | x | Describe the obstacles below! |

| Please describe the obstacles related to the technological resources or capacities. | If you are aware of any information source related to the obstacles, please, list here. |
| --- | --- |
| The technology used for outreach is the telephone. However, there are no dedicated phones for the purpose of contacting participants for screening, and the option to update information through the e-Health application is not utilized, as the application has not been fully accepted by citizens. |  |

**Question 7. Are there any major obstacles from the financing point of view to overcome the barrier?** (Mark your answer with an x.)

| No |  | Move to Question 8! |
| --- | --- | --- |
| Yes | x | Describe the obstacles below! |

| Please, describe the financial obstacles. | If you are aware of any information source related to the obstacles, please, list here. |
| --- | --- |
| No specific funds are allocated for these purposes.  Some health centers pay for their official phones, which are used for various functions, including screening outreach. In some cases, nurses use their personal phones for this purpose. While nurses are compensated as members of the selected gynecologist's team for providing services related to the screening program, they are dissatisfied with their remuneration. On the other hand, there is a lack of understanding among decision-makers regarding the need to increase financial compensation. |  |

**CHAPTER 3: STAKEHOLDERS’ PERSPECTIVES ON THE BARRIER**

**Question 8. Please, list all stakeholder groups who are affected by the barrier (i.e., those who have difficulties and obstacles due to the barrier)!**

| **Stakeholder group** | **Affected by the barrier?**  **(yes/no)** | **If yes, name the stakeholder(s)** | **If yes, define how they are affected?** |
| --- | --- | --- | --- |
| Ministries / regional or local governments | x | Ministry of health | Quality of screening |
| Public / private health insurance funds | x | Public insurance fund | Non-participation in the screening program leads to a higher incidence of cervical cancer among women, which in turn results in increased treatment costs for the disease. |
| Screening programme organizations | X | IPH | Quality indicators of screening |
| Other national institutes / committees / authorities |  |  |  |
| People eligible for screening (target population) | x |  | They are not invited to participate in screening |
| Medical professionals (including organizations) | x | Health center | They lack valid telephone numbers to reach the target population for screening, resulting in wasted time spent calling numbers that are outdated and no longer in use.  They have work obligations for which they are not sufficiently motivated to fulfill. |
| Health service providers (e.g., hospitals, laboratories) |  | Hospitals (Clinical Centar of Montenegro) | Due to the detection of disease at a later stage, there is an increased burden on healthcare facilities that provide treatment for patients. |
| Non-medical personnel in screening programme |  |  |  |
| International societies / organizations |  |  |  |
| Research institutes / experts |  |  |  |
| Patient organizations / Patient groups |  |  |  |
| Media / journalists |  |  |  |
| Other stakeholder groups (e.g., IT developers, NGOs) |  |  |  |

**Question 9. Please list all stakeholder groups who are capable to contribute to overcome the barrier!**

| **Stakeholder group** | **Capable to contribute to overcome?**  **(yes/no)** | **If yes, name the stakeholder(s)** | **If yes, define how and why they are capable?** |
| --- | --- | --- | --- |
| Ministries / regional or local governments | x | Ministry of health  Government of Montenegro | Making a decision/order that the provision of telephones for screening participants is of public health interest.  Resolving the staffing challenges for outreach can be accomplished by establishing new call centers, enhancing compensation and working conditions for those involved in the inviting oparticipant in the screening program, or by recruiting additional personnel. |
| Public / private health insurance funds | x |  | Increase compensation for contacting participants |
| Screening programme organizations | x | IPH | Suggest the solution |
| Other national institutes / committees / authorities |  |  |  |
| People eligible for screening (target population) |  |  |  |
| Medical professionals (including organizations) |  |  |  |
| Health service providers (e.g., hospitals, laboratories) |  |  |  |
| Non-medical personnel in screening programme |  |  |  |
| International societies / organizations |  |  |  |
| Research institutes / experts |  |  |  |
| Patient organizations / Patient groups |  |  |  |
| Media / journalists |  |  |  |
| Other stakeholder groups (e.g., IT developers, NGOs) |  |  |  |

**Question 10. Please list all stakeholder groups who does not support / are not motivated or interested / are opposing to overcome the barrier?**

| **Stakeholder group** | **Opposing to overcome?**  **(yes/no)** | **If yes, name the stakeholder(s)** | **If yes, define how and why they are opposing?** |
| --- | --- | --- | --- |
| Ministries / regional or local governments | X | Ministry of health  Government of Montenegro | Lack of understanding of the problem  Lack of will to solve the problem |
| Public / private health insurance funds |  |  |  |
| Screening programme organizations |  |  |  |
| Other national institutes / committees / authorities |  |  |  |
| People eligible for screening (target population) |  |  |  |
| Medical professionals (including organizations) |  |  |  |
| Health service providers (e.g., hospitals, laboratories) |  |  |  |
| Non-medical personnel in screening programme |  |  |  |
| International societies / organizations |  |  |  |
| Research institutes / experts |  |  |  |
| Patient organizations / Patient groups |  |  |  |
| Media / journalists |  |  |  |
| Other stakeholder groups (e.g., IT developers, NGOs) |  |  |  |

**CHAPTER 4: AVAILABLE KNOWLEDGE, DATA AND MONITORING OF THE BARRIER**

**Question 11. Can you identify any knowledge-based obstacles related to overcome the barrier (e.g., defining or updating guidelines or protocols)?** (Mark your answer with an x.)

| No | X | Move to Question 12! |
| --- | --- | --- |
| Yes |  | Describe the obstacles below! |

| Please, describe the knowledge-based obstacles. | If you are aware of any information source related to the obstacles, please, list here. |
| --- | --- |
|  |  |

**Question 12. Can you identify any obstacles related to cooperation and information sharing between the institutions with respect to overcome the barrier?** (Mark your answer with an x.)

| No | X | Move to Question 13! |
| --- | --- | --- |
| Yes |  | Describe the reason(s) below! |

| Please, describe the obstacles related to cooperation and information sharing. | If you are aware of any information source related to the obstacles, please, list here. |
| --- | --- |
|  |  |

**Question 13. Are there any relevant data or quantitative information available about the identified barrier?** (Mark your answer with an x.)

| No |  | Move to Question 14! |
| --- | --- | --- |
| Yes | x | Describe the reason(s) below! |

| Please, describe the available data. | If you are aware of any data, please, indicate the publicly available source here. |
| --- | --- |
| In the application of the screening program, all steps are noted and therefore measurable. There are defined indicators of the quality of the screening program. IPH publishes the Report on the implementation of the National  organized cervical cancer screening program  every year. The report contains all the necessary data. |  |

**Question 14. Are there any process, methods in place that can be used to monitor changes related to the barrier?** (Mark your answer with an x.)

| No |  | Move to the questions about the next barrier! |
| --- | --- | --- |
| Yes | X | Describe the reason(s) below! |

| Please, describe these processes, methods. | If you are aware of any information source related to the monitoring, please, list here. |
| --- | --- |
| Solving this barrier would significantly affect inviting women to participate in the screening program. The participation rate would also be higher. In the application of the screening program, all steps are noted and therefore measurable. There are defined indicators of the quality of the screening program. |  |

| **Barrier #2:** | Operation - Absence of an integrated screening registry for cervical cancer |
| --- | --- |

**CHAPTER 1: HISTORICAL CONTEXT OF THE IDENTIFIED BARRIER**

**Question 1. Can you identify any reason(s) responsible for the emergence of the barrier regarding the history of the screening program in your country?** (mark your answer with an x)

| No |  | Move to Question 2! |
| --- | --- | --- |
| Yes | x | Describe the reason(s) below! |

| Please, describe any historical reason that directly or indirectly led to the emergence of the barrier in the past (e.g., attributes, events).  *If there are multiple reasons, please describe all of them and define the most important one that led to the emergence of the barrier.* | If you are aware of any information source related to the reason(s), please, list here. |
| --- | --- |
| The main problem is the absence of a screening register. It is necessary to make a project proposal for the screening registry of cervical cancer.  Another issue is the use of two different data entry systems. Screening data is entered through the MG soft application, while data from Clinical Centar of MNE- gynecology (additional diagnostic procedures, surgical treatment), oncology (treatment) and pathology (cancer stage) are entered into Helliant, which is used in daily work. Due to the lack of staff, a large number of patients, gynecologists, oncologists and pathologists do not want to enter data twice through the Mg soft and Heliant application. Due to the lack of linkage between Heliant and the MG-Soft application, doctors from the Clinical Center of Montenegro are required to use both systems for screening purposes: Heliant is essential for documenting their daily work and invoicing provided services while MG-Soft is used for monitoring and evaluating the screening program. This insufficient connectivity between the two systems has posed a barrier since the begenning of the organized screening program.  IPH does not have access to that Heliant data. |  |

**Question 2. Are you aware of any activities or attempts that aimed to overcome the barrier in the past?** (mark your answer with an x)

| No |  | Move to Question 3! |
| --- | --- | --- |
| Yes | x | Describe the activities or attempts below! |

| Please, describe the activities and attempts that aimed to overcome the barrier in the past (i.e., how, when, by whom, where).  *Explain why these attempts failed / did not fully solve the problem.* | If you are aware of any information source related to the activities, please, list here. |
| --- | --- |
| The problem was repeatedly presented to the decision makers. The initiative has been launched multiple times by the Institute for Public Health of Montenegro. A working group was established to create a screening registry, and a study visit was organized for staff from IPH to learn about the functioning of the registry from colleagues in Slovenia. Unfortunately, the working group for establishing the registry is not currently operational |  |

**Question 3. Can you identify any relevant changes in the health care system or in other systems (political, economic or legislative) that effected the barrier in the past?** (mark your answer with an x)

| No |  | Move to Question 4! |
| --- | --- | --- |
| Yes | x | Describe the changes below! |

| Please, describe these changes that directly or indirectly affected the barrier.  *Explain whether the changes supported to overcome the barrier or made the problems more difficult.* | If you are aware of any information source related to the changes, please, list here. |
| --- | --- |
| In the previous few years, the minister of health and his associates have been changed several times. So we have again had to inform the decision makers about the barriers in the screening program. |  |

**CHAPTER 2: CAPABILITIES AND RESOURCES THAT INFLUENCE THE BARRIER**

**Question 4. Does the current legislative, political and economic environment allow or support to overcome the identified barrier?** (mark your answer with an x)

| Yes | x | Move to Question 5! |
| --- | --- | --- |
| No |  | Describe the reason(s) below! |

| Please, describe any reason from the legislative, political and economic environment point of view that does not allow or support to overcome the identified barrier.  *If there are multiple reasons, please, describe all of them and define the most important one.* | If you are aware of any information source related to the reason(s), please, list here. |
| --- | --- |
|  |  |

**Question 5. Are there any major obstacles from the human resource point of view to overcome the barrier?** (mark your answer with an x)

| No | x | Move to Question 6! |
| --- | --- | --- |
| Yes |  | Describe the obstacles below! |

| Please, describe the human resource obstacles. | If you are aware of any information source related to the obstacles, please, list here. |
| --- | --- |
|  |  |

**Question 6. Are there any major obstacles from the technological resources or capacities point of view to overcome the barrier? (IT system, screening technologies and devices, equipment etc.)** (mark your answer with an x)

| No |  | Move to Question 7! |
| --- | --- | --- |
| Yes | x | Describe the obstacles below! |

| Please describe the obstacles related to the technological resources or capacities. | If you are aware of any information source related to the obstacles, please, list here. |
| --- | --- |
| Currently, there is no way to connect data that is in two different systems (Mg soft and Heliant). They function independently of each other. It is necessary to extract the data found in Heliant (gynecologists, pathologists, oncologists use it for daily work and screening) and fill the database used by Mg soft. |  |

**Question 7. Are there any major obstacles from the financing point of view to overcome the barrier?** (mark your answer with an x)

| No |  | Move to Question 8! |
| --- | --- | --- |
| Yes | X | Describe the obstacles below! |

| Please, describe the financial obstacles. | If you are aware of any information source related to the obstacles, please, list here. |
| --- | --- |
| Significant financial resources are necessary. It isn’t provided in the cervical cancer screening budget. |  |

**CHAPTER 3: STAKEHOLDERS’ PERSPECTIVES ON THE BARRIER**

**Question 8. Please, list all stakeholder groups who are affected by the barrier (i.e., those who have difficulties and obstacles due to the barrier)!**

| **Stakeholder group** | **Affected by the barrier?**  **(yes/no)** | **If yes, name the stakeholder(s)** | **If yes, define how they are affected?** |
| --- | --- | --- | --- |
| Ministries / regional or local governments | X | Ministry of health | Quality od screening program, inadequate data |
| Public / private health insurance funds |  |  |  |
| Screening programme organizations | x | IPH | Inadequate data for monitoring of screening program |
| Other national institutes / committees / authorities |  |  |  |
| People eligible for screening (target population) |  |  |  |
| Medical professionals (including organizations) |  |  |  |
| Health service providers (e.g. hospitals, laboratories) |  |  |  |
| Non-medical personnel in screening programme |  |  |  |
| International societies / organizations |  |  |  |
| Research institutes / experts | X |  | Lack of data |
| Patient organizations / Patient groups |  |  |  |
| Media / journalists |  |  |  |
| Other stakeholder groups (e.g. IT developers, NGOs) |  |  |  |

**Question 9. Please list all stakeholder groups who are capable to contribute to overcome the barrier!**

| **Stakeholder group** | **Capable to contribute to overcome?**  **(yes/no)** | **If yes, name the stakeholder(s)** | **If yes, define how and why they are capable?** |
| --- | --- | --- | --- |
| Ministries / regional or local governments | x | Ministry of health | Decision on establishing a screening registry and hiring staff. Providing financial resources for integration of two systems (Mg soft and Heliant) |
| Public / private health insurance funds | x | Public health insurance fund | Financial resources |
| Screening programme organizations | x | IPH | Defining a set of data that is necessary for monitoring the screening program |
| Other national institutes / committees / authorities | X | Clinical center of Montenegro | Necessary consent for integration of 2 systems |
| People eligible for screening (target population) |  |  |  |
| Medical professionals (including organizations) |  |  |  |
| Health service providers (e.g. hospitals, laboratories) |  |  |  |
| Non-medical personnel in screening programme |  |  |  |
| International societies / organizations |  |  |  |
| Research institutes / experts |  |  |  |
| Patient organizations / Patient groups |  |  |  |
| Media / journalists |  |  |  |
| Other stakeholder groups (e.g. IT developers, NGOs) | x | Helliant, Mg soft | Find the solution, co-operation |

**Question 10. Please list all stakeholder groups who does not support / are not motivated or interested / are opposing to overcome the barrier?**

| **Stakeholder group** | **Opposing to overcome?**  **(yes/no)** | **If yes, name the stakeholder(s)** | **If yes, define how and why they are opposing?** |
| --- | --- | --- | --- |
| Ministries / regional or local governments | X | Ministry of health | Lack of will to solve problem |
| Public / private health insurance funds |  |  |  |
| Screening programme organizations |  |  |  |
| Other national institutes / committees / authorities |  |  |  |
| People eligible for screening (target population) |  |  |  |
| Medical professionals (including organizations) |  |  |  |
| Health service providers (e.g. hospitals, laboratories) |  |  |  |
| Non-medical personnel in screening programme |  |  |  |
| International societies / organizations |  |  |  |
| Research institutes / experts |  |  |  |
| Patient organizations / Patient groups |  |  |  |
| Media / journalists |  |  |  |
| Other stakeholder groups (e.g. IT developers, NGOs) | X | Helliant, Mg soft |  |

**CHAPTER 4: AVAILABLE KNOWLEDGE, DATA AND MONITORING OF THE BARRIER**

**Question 11. Can you identify any knowledge-based obstacles related to overcome the barrier (e.g., defining or updating guidelines or protocols)?** (mark your answer with an x)

| No |  | Move to Question 12! |
| --- | --- | --- |
| Yes | X | Describe the obstacles below! |

| Please, describe the knowledge-based obstacles. | If you are aware of any information source related to the obstacles, please, list here. |
| --- | --- |
| It is essential to define a dataset for the screening registry |  |

**Question 12. Can you identify any obstacles related to cooperation and information sharing between the institutions with respect to overcome the barrier?** (mark your answer with an x)

| No |  | Move to Question 13! |
| --- | --- | --- |
| Yes | X | Describe the reason(s) below! |

| Please, describe the obstacles related to cooperation and information sharing. | If you are aware of any information source related to the obstacles, please, list here. |
| --- | --- |
| Helliant and Mg soft don't want to cooperate. |  |

**Question 13. Are there any relevant data or quantitative information available about the identified barrier?** (mark your answer with an x)

| No |  | Move to Question 14! |
| --- | --- | --- |
| Yes | x | Describe the reason(s) below! |

| Please, describe the available data. | If you are aware of any data, please, indicate the publicly available source here. |
| --- | --- |
| IPH publishes the Report on the implementation of the National organized cervical cancer screening program every year. The report contains all the necessary data, but it does not include data concerning surgery, treatment and follow-up. |  |

**Question 14. Are there any process, methods in place that can be used to monitor changes related to the barrier?** (mark your answer with an x)

| No |  | Move to the questions about the next barrier! |
| --- | --- | --- |
| Yes | x | Describe the reason(s) below! |

| Please, describe these processes, methods. | If you are aware of any information source related to the monitoring, please, list here. |
| --- | --- |
| By overcoming this barrier, the necessary data will be available, the quality of monitoring will be significantly improved, and the annual report on the implementation of the screening program will contain all the necessary data. |  |

| **Barrier #3:** | Knowledge - Lack of strategy for prevention and early detection of cervical cancer |
| --- | --- |

**CHAPTER 1: HISTORICAL CONTEXT OF THE IDENTIFIED BARRIER**

**Question 1. Can you identify any reason(s) responsible for the emergence of the barrier regarding the history of the screening program in your country?** (mark your answer with an x)

| No |  | Move to Question 2! |
| --- | --- | --- |
| Yes | X | Describe the reason(s) below! |

| Please, describe any historical reason that directly or indirectly led to the emergence of the barrier in the past (e.g., attributes, events).  *If there are multiple reasons, please describe all of them and define the most important one that led to the emergence of the barrier.* | If you are aware of any information source related to the reason(s), please, list here. |
| --- | --- |
| In recent years, there have been several changes in the Minister of Health and his associates. As a result, we have had to repeatedly inform decision-makers about the barriers affecting the screening program. The lack of continuity in the management structures within the Ministry of Health makes it challenging to develop any plans, strategies, or documents. A strategy for the development of the screening program has never been established. Although national programs exist, they are from 2011 and have not been updated since. |  |

**Question 2. Are you aware of any activities or attempts that aimed to overcome the barrier in the past?** (mark your answer with an x)

| No |  | Move to Question 3! |
| --- | --- | --- |
| Yes | x | Describe the activities or attempts below! |

| Please, describe the activities and attempts that aimed to overcome the barrier in the past (i.e., how, when, by whom, where).  *Explain why these attempts failed / did not fully solve the problem.* | If you are aware of any information source related to the activities, please, list here. |
| --- | --- |
| There is a working version of the law on population health care and strategies and programs for the prevention and control of non-communicable diseases, but it has not yet been adopted. There is no specific strategy for cervical cancer screening, and the program is not currently valid. |  |

**Question 3. Can you identify any relevant changes in the health care system or in other systems (political, economic or legislative) that effected the barrier in the past?** (mark your answer with an x)

| No |  | Move to Question 4! |
| --- | --- | --- |
| Yes | X | Describe the changes below! |

| Please, describe these changes that directly or indirectly affected the barrier.  *Explain whether the changes supported to overcome the barrier or made the problems more difficult.* | If you are aware of any information source related to the changes, please, list here. |
| --- | --- |
| In the previous few years, the minister of health and his associates have been changed several times. |  |

**CHAPTER 2: CAPABILITIES AND RESOURCES THAT INFLUENCE THE BARRIER**

**Question 4. Does the current legislative, political and economic environment allow or support to overcome the identified barrier?** (mark your answer with an x)

| Yes | X | Move to Question 5! |
| --- | --- | --- |
| No |  | Describe the reason(s) below! |

| Please, describe any reason from the legislative, political and economic environment point of view that does not allow or support to overcome the identified barrier.  *If there are multiple reasons, please, describe all of them and define the most important one.* | If you are aware of any information source related to the reason(s), please, list here. |
| --- | --- |
|  |  |

**Question 5. Are there any major obstacles from the human resource point of view to overcome the barrier?** (mark your answer with an x)

| No | X | Move to Question 6! |
| --- | --- | --- |
| Yes |  | Describe the obstacles below! |

| Please, describe the human resource obstacles. | If you are aware of any information source related to the obstacles, please, list here. |
| --- | --- |
|  |  |

**Question 6. Are there any major obstacles from the technological resources or capacities point of view to overcome the barrier? (IT system, screening technologies and devices, equipment etc.)** (mark your answer with an x)

| No | X | Move to Question 7! |
| --- | --- | --- |
| Yes |  | Describe the obstacles below! |

| Please describe the obstacles related to the technological resources or capacities. | If you are aware of any information source related to the obstacles, please, list here. |
| --- | --- |
|  |  |

**Question 7. Are there any major obstacles from the financing point of view to overcome the barrier?** (mark your answer with an x)

| No | X | Move to Question 8! |
| --- | --- | --- |
| Yes |  | Describe the obstacles below! |

| Please, describe the financial obstacles. | If you are aware of any information source related to the obstacles, please, list here. |
| --- | --- |
|  |  |

**CHAPTER 3: STAKEHOLDERS’ PERSPECTIVES ON THE BARRIER**

**Question 8. Please, list all stakeholder groups who are affected by the barrier (i.e., those who have difficulties and obstacles due to the barrier)!**

| **Stakeholder group** | **Affected by the barrier?**  **(yes/no)** | **If yes, name the stakeholder(s)** | **If yes, define how they are affected?** |
| --- | --- | --- | --- |
| Ministries / regional or local governments | X | Ministry of health | A strategy for the development of screening programs is important for the Ministry of Health because it improves public health outcomes through early disease detection, allows for better resource allocation, ensures continuity despite leadership changes, enables data-driven decision-making, increases public awareness and participation, aligns with national and international guidelines, and facilitates evaluation and improvement of the program. |
| Public / private health insurance funds | x | Public health insurance funds | Treatment costs for cancer |
| Screening programme organizations | X | IPH | A strategy for screening programs is important for the Institute of Public Health because it enables effective coordination among stakeholders, enhances monitoring and evaluation of outcomes, facilitates systematic data collection, optimizes resource use, and ultimately contributes to improved public health outcomes |
| Other national institutes / committees / authorities |  |  |  |
| People eligible for screening (target population) |  |  |  |
| Medical professionals (including organizations) | X | Gynecologist |  |
| Health service providers (e.g. hospitals, laboratories) | X | Health centers, general hospitals |  |
| Non-medical personnel in screening programme |  |  |  |
| International societies / organizations |  |  |  |
| Research institutes / experts |  |  |  |
| Patient organizations / Patient groups |  |  |  |
| Media / journalists |  |  |  |
| Other stakeholder groups (e.g. IT developers, NGOs) |  |  |  |

**Question 9. Please list all stakeholder groups who are capable to contribute to overcome the barrier!**

| **Stakeholder group** | **Capable to contribute to overcome?**  **(yes/no)** | **If yes, name the stakeholder(s)** | **If yes, define how and why they are capable?** |
| --- | --- | --- | --- |
| Ministries / regional or local governments | X | Ministry of health  Government of Montenegro | The dynamics of the creation of strategic documents.  Adoption of documents. |
| Public / private health insurance funds |  |  |  |
| Screening programme organizations |  |  |  |
| Other national institutes / committees / authorities |  |  |  |
| People eligible for screening (target population) |  |  |  |
| Medical professionals (including organizations) |  |  |  |
| Health service providers (e.g. hospitals, laboratories) |  |  |  |
| Non-medical personnel in screening programme |  |  |  |
| International societies / organizations |  |  |  |
| Research institutes / experts |  |  |  |
| Patient organizations / Patient groups |  |  |  |
| Media / journalists |  |  |  |
| Other stakeholder groups (e.g. IT developers, NGOs) |  |  |  |

**Question 10. Please list all stakeholder groups who does not support / are not motivated or interested / are opposing to overcome the barrier?**

| **Stakeholder group** | **Opposing to overcome?**  **(yes/no)** | **If yes, name the stakeholder(s)** | **If yes, define how and why they are opposing?** |
| --- | --- | --- | --- |
| Ministries / regional or local governments | X | Ministry of health | The slowness of creating strategic documents |
| Public / private health insurance funds |  |  |  |
| Screening programme organizations |  |  |  |
| Other national institutes / committees / authorities |  |  |  |
| People eligible for screening (target population) |  |  |  |
| Medical professionals (including organizations) |  |  |  |
| Health service providers (e.g. hospitals, laboratories) |  |  |  |
| Non-medical personnel in screening programme |  |  |  |
| International societies / organizations |  |  |  |
| Research institutes / experts |  |  |  |
| Patient organizations / Patient groups |  |  |  |
| Media / journalists |  |  |  |
| Other stakeholder groups (e.g. IT developers, NGOs) |  |  |  |

**CHAPTER 4: AVAILABLE KNOWLEDGE, DATA AND MONITORING OF THE BARRIER**

**Question 11. Can you identify any knowledge-based obstacles related to overcome the barrier (e.g., defining or updating guidelines or protocols)?** (mark your answer with an x)

| No | X | Move to Question 12! |
| --- | --- | --- |
| Yes |  | Describe the obstacles below! |

| Please, describe the knowledge-based obstacles. | If you are aware of any information source related to the obstacles, please, list here. |
| --- | --- |
|  |  |

**Question 12. Can you identify any obstacles related to cooperation and information sharing between the institutions with respect to overcome the barrier?** (mark your answer with an x)

| No | X | Move to Question 13! |
| --- | --- | --- |
| Yes |  | Describe the reason(s) below! |

| Please, describe the obstacles related to cooperation and information sharing. | If you are aware of any information source related to the obstacles, please, list here. |
| --- | --- |
|  |  |

**Question 13. Are there any relevant data or quantitative information available about the identified barrier?** (mark your answer with an x)

| No | X | Move to Question 14! |
| --- | --- | --- |
| Yes |  | Describe the reason(s) below! |

| Please, describe the available data. | If you are aware of any data, please, indicate the publicly available source here. |
| --- | --- |
|  |  |

**Question 14. Are there any process, methods in place that can be used to monitor changes related to the barrier?** (mark your answer with an x)

| No | X | Move to the questions about the next barrier! |
| --- | --- | --- |
| Yes |  | Describe the reason(s) below! |

| Please, describe these processes, methods. | If you are aware of any information source related to the monitoring, please, list here. |
| --- | --- |
|  |  |

**Final question of the document**

Can you name any further stakeholder(s) of the screening program who would also be capable to answer these questions and who could provide valuable information about these barriers from a stakeholder perspective different from yours?

Please name this/these stakeholder(s) and provide contact information below!

| Ministry of health |
| --- |

Background information

Please name the country for which you complete this document!

| Albania |
| --- |

Please name the cancer site for which you complete this document!

| Cervical Cancer |
| --- |

Please list the most important barriers that were identified in the Barrier Assessment Tool!

| Barrier #1: | Issue with establishing protocols, procedures and legal framework (opportunistic screening) |
| --- | --- |
| Barrier #2: | Insufficient human, physical and/or financial recourses to operate screening program |
| Barrier #3: | Inadequate system for monitoring treatment information |

Please describe your position or your relationship with the screening programme (programme coordinator / researcher / policymaker / clinician / patient)!

| Programme management / researcher / |
| --- |

Please mark the date when you completed this barrier assessment document (year and months)!

| November 2023 |
| --- |

**CHAPTER 1: HISTORICAL CONTEXT OF THE IDENTIFIED BARRIERS**

**Question 1. Can you identify any reason(s) responsible for the emergence of the barriers regarding the history of the screening program in your country?**(mark your answer with an x)

| No |  | Move to Question 2! |
| --- | --- | --- |
| **Yes** |  | Describe the reason(s) below! x |

| Please, describe any historical reason that directly or indirectly led to the emergence of the barrier in the past (e.g.,attributes, events).  *If there are multiple reasons, please describe all of them and define the most important one that led to the emergence of the barrier.* | If you are aware of any information source related to the reason(s), please, list here. |
| --- | --- |
| 1. There was a persistent perception among political leaders and health care decision-makers that HPV testing was too expensive and this perception was fostered by a limited understanding of the poor performance and true costs of VIA and cytology. 2. Medical professional also had a limited understanding of the benefits and drawbacks of the different screening tests, which for some time led to a lack of consensus about implementing HPV testing | No documents about it. Perception based on the 8 years efforts to establish the screening program |

**Question 2. Are you aware of any activities or attempts that aimed to overcome the barriers in the past?**(mark your answer with an x)

| No |  | Move to Question 3! |
| --- | --- | --- |
| Yes |  | Describe the activities or attempts below!x |

| Please, describe the activities and attempts that aimed to overcome the barrier in the past (i.e., how, when, by whom, where).  *Explain why these attempts failed / did not fully solve the problem.* | If you are aware of any information source related to the activities, please, list here. |
| --- | --- |
| National Conferences  Consensus building workshops, including UN and EU-TAIEX supported activities  Policy brief  Cost-effectivity of HPV screening program |  |

**Question 3. Can you identify any relevant changes in the health care system or in other systems (political, economic or legislative) that effected the barriers in the past?**(mark your answer with an x)

| No |  | Move to Question 4! |
| --- | --- | --- |
| Yes |  | Describe the changes below!x |

| Please, describe these changes that directly or indirectly affected the barrier.  *Explain whether the changes supported to overcome the barrier or made the problems more difficult.* | If you are aware of any information source related to the changes, please, list here. |
| --- | --- |
| New legal frame for screening helped overcoming all resistance in health sector | VKM 2019  Legal low of Prime Minister for cervical cancer screening |

**CHAPTER 2: CAPABILITIES AND RESOURCES THAT INFLUENCE THE BARRIERS**

**Question 4. Does the current legislative, political and economic environment allow or support to overcome the identified barriers?**(mark your answer with an x)

| yes | x | Move to Question 5! |
| --- | --- | --- |
| No |  | Describe the reason(s) below! |

| Please, describe any reason from the legislative, political and economic environment point of view that does not allow or support to overcome the identified barrier.  *If there are multiple reasons, please, describe all of them and define the most important one.* | If you are aware of any information source related to the reason(s), please, list here. |
| --- | --- |
|  |  |

**Question 5. Are there any major obstacles from the human resource point of view to overcome the barriers?** (mark your answer with an x)

| No |  | Move to Question 6! |
| --- | --- | --- |
| Yes | x | Describe the obstacles below! |

| Please, describe the human resource obstacles. | If you are aware of any information source related to the obstacles, please, list here. |
| --- | --- |
| The existing structures at national HPV laboratory, central and local program management are insufficient.  Capacities for diagnoses (colposcopy) and treatment are limited and there are no systems for quality assurance and continuous medical training |  |

**Question 6. Are there any major obstacles from the technological resources or capacities point of view to overcome the barriers? (IT system, screening technologies and devices, equipment etc.)**(mark your answer with an x)

| No |  | Move to Question 7! |
| --- | --- | --- |
| Yes | x | Describe the obstacles below! |

| Please describe the obstaclesrelated to the technological resources or capacities. | If you are aware of any information source related to the obstacles, please, list here. |
| --- | --- |
| Monitoring systems covers only primary testing and not the follow up.  Individual forms are not automatized  IT system needed |  |

**Question 7. Are there any major obstacles from the financing point of view to overcome the barriers?**(mark your answer with an x)

| No |  | Move to Question 8! |
| --- | --- | --- |
| Yes | X | Describe the obstacles below! |

| Please, describe the financial obstacles. | If you are aware of any information source related to the obstacles, please, list here. |
| --- | --- |
| Budget for procuring HPV test consumables are limited to the age group of 40-50 and only to the coverage 30%.  Budget for logistics are insufficient and are only partially absorbed by existing resources of public health authorities |  |

**CHAPTER 3: STAKEHOLDERS’ PERSPECTIVES ON THE BARRIERS**

**Question 8. Please, list all stakeholder groups who are affected by the barrier (i.e., those who have difficulties and obstacles due to the barrier)!**

| **Stakeholder group** | **Affected by the barrier?**  **(yes/no)** | **If yes, name the stakeholder(s)** | **If yes, define how they are affected?** |
| --- | --- | --- | --- |
| Ministries / regional or local governments | x | MOH, Primary health care | Increased budget, legal framework |
| Public / private health insurance funds |  |  |  |
| Screening programme organizations | x | IPH, and structures | Collaboration and reporting, and IT |
| Other national institutes / committees / authorities | x | Primary health care center, Maternity, University health center | Burden work, and quality and |
| People eligible for screening (target population) | x | Increasing target group screening | Target screening coverage |
| Medical professionals (including organizations) | x | Training all part of program | All structures |
| Health service providers (e.g. hospitals, laboratories) | x | Training of personnel, furniture, and coding procedures | Loosing time, quality, and precision. Data bank |
| Non-medical personnel in screening programme | x | psychologist | Not be part of multidisciplinary treatment and support for patients |
| International societies / organizations |  |  |  |
| Research institutes / experts | x | IPH | Missing reporting of follow-up, |
| Patient organizations / Patient groups | x | Europe Dona part of ENGAGE | Missing information |
| Media / journalists |  |  |  |
| Other stakeholder groups (e.g. IT developers, NGOs) |  |  |  |

**Question 9. Please list all stakeholder groups who are capable to contribute to overcome the barriers!**

| **Stakeholder group** | **Capable to contribute to overcome?**  **(yes/no)** | **If yes, name the stakeholder(s)** | **If yes, define how and why they are capable?** |
| --- | --- | --- | --- |
| Ministries / regional orlocal governments | x | MOH, Primary health care | Organize work |
| Public / private health insurance funds | x | Privet sector | Reporting |
| Screening programme organizations | x | IPH, and structures |  |
| Other national institutes / committees / authorities | x | Primary health care center, Maternity, University health center |  |
| People eligible for screening (target population) | x | Increasing target group screening |  |
| Medical professionals (including organizations) | x | Training all part of program |  |
| Health service providers (e.g. hospitals, laboratories) | x | Training of personnel, furniture, and coding procedures |  |
| Non-medical personnel in screening programme | x | psychologist |  |
| International societies / organizations | x |  |  |
| Research institutes / experts | x | IPH |  |
| Patient organizations / Patient groups | x | Europe Dona part of ENGAGE |  |
| Media / journalists | x |  |  |
| Other stakeholder groups (e.g. IT developers, NGOs) | x |  |  |

**Question 10. Please list all stakeholder groups who does not support / are not motivated or interested / are opposing to overcome the barriers?**

| **Stakeholder group** | **Opposing to overcome?**  **(yes/no)** | **If yes, name the stakeholder(s)** | **If yes, define how and why they are opposing?** |
| --- | --- | --- | --- |
| Ministries / regional orlocal governments |  |  |  |
| Public / private health insurance funds | No |  |  |
| Screening programme organizations |  |  |  |
| Other national institutes / committees / authorities |  |  |  |
| People eligible for screening (target population) |  |  |  |
| Medical professionals (including organizations) |  |  |  |
| Health service providers (e.g. hospitals, laboratories) |  |  |  |
| Non-medical personnel in screening programme |  |  |  |
| International societies / organizations |  |  |  |
| Research institutes / experts |  |  |  |
| Patient organizations / Patient groups |  |  |  |
| Media / journalists |  |  |  |
| Other stakeholder groups (e.g. IT developers, NGOs) |  |  |  |

**CHAPTER 4: AVAILABLE KNOWLEDGE, DATA AND MONITORING OF THE BARRIERS**

**Question 11. Can you identify any knowledge-based obstacles related to overcome the barriers (e.g., defining or updating guidelines or protocols)?**(mark your answer with an x)

| No |  | Move to Question 12! |
| --- | --- | --- |
| Yes | x | Describe the obstacles below! |

| Please, describe the knowledge-based obstacles. | If you are aware of any information source related to the obstacles, please, list here. |
| --- | --- |
| Professional society, Fund health insurance, Primary health Care, Public Health, Promotion collaboration | Update on guideline and protocols of screening |

**Question 12. Can you identify any obstacles related tocooperation and information sharing between the institutions with respect to overcome the barriers?**(mark your answer with an x)

| No |  | Move to Question 13! |
| --- | --- | --- |
| Yes |  | Describe the reason(s) below! |

| Please, describe the obstacles related to cooperation and information sharing. | If you are aware of any information source related to the obstacles, please, list here. |
| --- | --- |
| Collaboration between institution and reporting case in diagnose and treatment of cervical cancer patients and preneoplasica lesions Cin 1-Cin3 | Reporting case and IT information |

**Question 13. Arethere any relevant data or quantitative information available about the identified barriers?**(mark your answer with an x)

| No | x | Move to Question 14! |
| --- | --- | --- |
| Yes |  | Describe the reason(s) below! |

| Please, describe the available data. | If you are aware of any data, please, indicate the publicly available source here. |
| --- | --- |
|  |  |

**Question 14. Are there any process, methods in place that can be used to monitor changes related to the barriers?** (mark your answer with an x)

| No |  | Move to the questions about the next barrier! |
| --- | --- | --- |
| Yes | x | Describe the reason(s) below! |

| Please, describe these processes, methods. | If you are aware of any information source related to the monitoring, please, list here. |
| --- | --- |
| Analyzing data and prepare report annual | All the institution that are working on cervical cancer |

**Final question of the document**

Can you name any further stakeholder(s)of the screening program who would also be capable to answer these questions and who could provide valuable information about these barriers from a stakeholder perspective different from yours?

Please name this/these stakeholder(s) and provide contact information below!

| Maternity and Oncological Hospital |
| --- |
